# Factors Produced by Macrophages Eliminating Apoptotic Cells Demonstrate Pro-Resolutive Properties and Terminate Ongoing Inflammation

**DOI:** 10.3389/fimmu.2018.02586

**Published:** 2018-11-13

**Authors:** Francis Bonnefoy, Thierry Gauthier, Romain Vallion, Omayra Martin-Rodriguez, Anais Missey, Anna Daoui, Séverine Valmary-Degano, Philippe Saas, Mélanie Couturier, Sylvain Perruche

**Affiliations:** ^1^INSERM, EFS Bourgogne Franche-Comté, UMR1098, Interactions Hôte-Greffon-Tumeur, LabEX LipSTIC, FHU INCREASE, Université Bourgogne Franche-Comté, Besançon, France; ^2^Department of Pathology, Besancon University Hospital, Besançon, France; ^3^MED'INN'Pharma, Besançon, France

**Keywords:** efferocytosis, apoptotic cells, resolution of inflammation, macrophages, phagocytosis, tolerance induction

## Abstract

Unresolved inflammation is a common feature in the pathogenesis of chronic inflammatory/autoimmune diseases. The factors produced by macrophages eliminating apoptotic cells during resolution are crucial to terminate inflammation, and for subsequent tissue healing. We demonstrated here that the factors produced by macrophages eliminating apoptotic cells were sufficient to reboot the resolution of inflammation *in vivo*, and thus definitively terminated ongoing chronic inflammation. These factors were called SuperMApo and revealed pro-resolutive properties and accelerated acute inflammation resolution, as attested by both increased phagocytic capacities of macrophages and enhanced thioglycollate-induced peritonitis resolution. Activated antigen-presenting cells exposed to SuperMApo accelerated their return to homeostasis and demonstrated pro-regulatory T cell properties. In mice with ongoing collagen-induced arthritis, SuperMApo injection resolved and definitively terminated chronic inflammation. The same pro-resolving properties were observed in human settings in addition to xenogeneic colitis and graft-*vs*.-host disease modulation, highlighting SuperMApo as a new therapeutic opportunity to circumvent inflammatory diseases.

## Introduction

Inflammation is a natural reaction of the body to fight and control a threat like infections or injuries. The inflammatory response under homeostatic conditions is well-orchestrated and self-resolved (1). However, due to genetic abnormalities, chronic exposure to environmental agents or a persistent disease, resolution could be dysregulated triggering chronic inflammation/autoimmunity (2–4). Whereas, efferocytosis plays a major role in the resolution and may represent therapeutic opportunities (2, 5–7), the use of the whole range of factors issued from efferocytosis to trigger resolution and terminate inflammation has not been addressed.

During inflammation, blood polymorphonuclear cells (PMN) infiltrate the targeted tissue followed by monocytes. This infiltrate controls and eliminates the threat (8). Then, probably initiated by lipids, converted from pro-inflammatory to specialized pro-resolutive mediators, resolution takes place, with the clearance of dead cells and debris by tissue-resident phagocytes and/or monocyte-derived macrophages. Resolution terminates inflammation (9). Resolution depends on the efficient elimination of apoptotic cells, i.e., efferocytosis, (10) to prevent the release of damage-associated molecular patterns (DAMPs), and on the secretion of anti-inflammatory and pro-resolutive factors by both apoptotic cells and macrophages eliminating apoptotic cells (11, 12). In addition to prevent DAMP triggering chronic inflammation (13, 14), resolution restores homeostasis (15) and initiates tissue healing (16).

Efferocytosis provides therefore a complete and complex “resolutome” (i.e., a set of proteins and lipids notably) able to terminate inflammation and initiate healing; here, we demonstrate that indeed the use of these factors issued from efferocytosis taken as a whole, resolves acute and chronic inflammation. The factors within the *super*natant of *m*acrophages eliminating *apo*ptotic cells (called SuperMApo) demonstrated pro-resolutive properties *in vitro* and in experimental models of inflammation, by affecting neutrophils, plasmacytoid dendritic cells (pDC), macrophages and CD4^+^ regulatory T cells (Treg). Remarkably, SuperMApo reprogrammed macrophages to acquire pro-Treg properties *in vitro* and in the collagen-induced arthritis (CIA) model where SuperMApo injection resolved ongoing arthritis. We observed that associated with other factors, TGF-β within SuperMApo played a critical role in resolution. SuperMApo from human cells also demonstrated pro-resolutive properties *in vitro* and in peritonitis, colitis and graft-*vs*.-host disease xenogeneic models. Our data demonstrate that the factors issued from efferocytosis, i.e., SuperMApo, allow the resolution of ongoing inflammation, and strongly support the use of SuperMApo as an innovative therapeutic approach to circumvent inflammatory diseases.

## Results

### The factors issued from apoptotic cell elimination by macrophages demonstrate pro-resolutive properties

**Figure 1:**
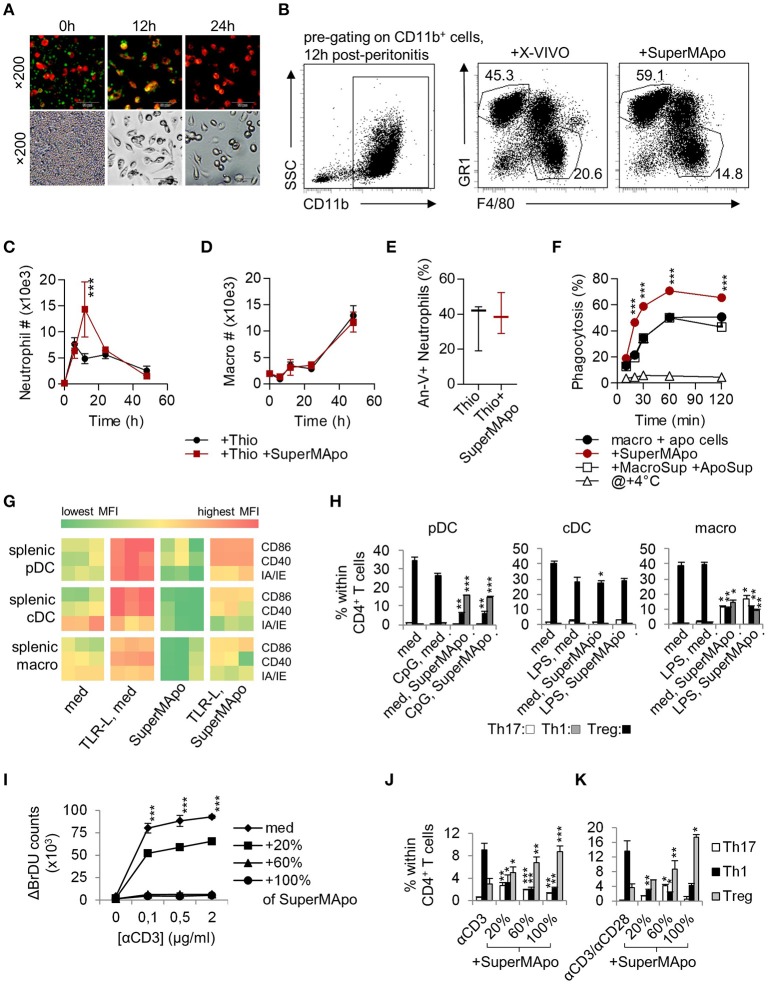
Macrophages eliminating apoptotic cells produced factors with pro-resolutive properties. **(A)** Inverted fluorescence microscopy observation of macrophages stained by PE-F4/80 antibody (red), eliminating CFSE-labeled apoptotic cells (green) at 0, 12, and 24 h of culture. Injection (i.p.) of the supernatant from the previous 48 h culture (SuperMApo) increased neutrophil percentage **(B)** and number **(C)**, not macrophage number **(D)** in the peritoneal cavity after peritonitis induction. **(E)** Neutrophil apoptosis 24 h after peritonitis induction in the presence or not of SuperMApo injection 28 h earlier (*n* = 3 mice per group; box and whiskers). The gating strategy of peritoneal neutrophils and macrophages is shown in **(B)**, other data are shown as mean ± s.e.m., *n* = 3. ***P < 0.001, two-way RM ANOVA with Bonferroni post-tests. **(F)** The percentage of phagocytosis (mean ± s.e.m., *n* = 3) of apoptotic cells by macrophages was evaluated at various time points in normal culture condition (macro + apo cells) with or without SuperMApo or supernatant from macrophages cultured alone plus the supernatant from apoptotic cells cultured alone (+MacroSup +ApoSup), or at +4°C. ***P < 0.001, two-way RM ANOVA with Bonferroni post-tests. **(G)** Costimulatory (CD86/CD40) and MHC-II molecule (IA/IE) mean florescence intensity (MFI) expressions evaluated in plasmacytoid DC (pDC), conventional DC (cDC) and macrophages (macro) in spleen cell cultures with or without TLR ligands (TLR-L) or SuperMApo, or medium (med). Each cell in the heat map represents a single well; data are from one experiment representative of three. (**H**) Ovalbumin (OVA) TCR-specific CD4^+^CD25^−^ T cell polarization (mean ± s.e.m., *n* = .3) by pDC, cDC and macrophages cultured as in **G** in the presence of OVA, was assessed by FACS evaluating IFN-γ (Th1), IL-17 (Th17) and Foxp3 (Treg) intracellular content after 4 days of culture. *P < 0.05, **P < 0.01, ***P < 0.001, 1 way ANOVA with Bonferroni's multiple comparison test. **(I)** Spleen T cell proliferation in the presence of grading doses of anti-CD3 specific antibody (αCD3) with medium (med) or SuperMApo in different proportions was assessed by BrdU incorporation and counting. ***P < 0.001, med vs. other conditions (mean ± s.e.m., *n* = 3), two-way RM ANOVA with Tukey's multiple comparison test. CD4 T cell polarization within spleen cells cultured as in **I (J)**, or from naïve CD4^+^CD25^−^ T cells cultured with anti-CD3/CD28 antibodies **(K)** in medium (med) or different proportions of SuperMApo was assessed by FACS. *P < 0.05, **P < 0.01, ***P < 0.001, unpaired *t*-test vs. without SuperMApo (mean ± s.e.m., *n* = 3). Figure data are issued from representative experiments, repeated at least three times with similar results.

Peritoneal-sorted macrophages, elicited by thioglycollate, were very efficient to eliminate apoptotic cells in 24 h (Figure 1A) and the culture supernatant (i.e., SuperMApo) was collected 24 h later. SuperMApo injection in the model of thioglycollate-induced peritonitis enhanced resolution increasing the influx of neutrophils 12 h after induction (Figures 1B,C). Because apoptosis of neutrophils at 24 h and numbers of macrophages were found similar (Figures 1D,E), the data demonstrated that SuperMApo injection enhanced as well the capacity of macrophages to eliminate 2 to 3 time more apoptotic neutrophils since similar numbers of neutrophils were found between conditions at 24 h (Figures 1B,C). Enhanced phagocytic capacity of macrophages was confirmed *in vitro* in the presence of SuperMApo where they demonstrated greater phagocytic capacities to eliminate apoptotic cells (Figure 1F), as well as beads or bacteria (not shown). Interestingly, this experiment also demonstrated that the factors issued from cultured apoptotic cells or those from cultured macrophages did not enhanced efferocytosis in culture (Figure 1F). So far, efferocytosis factors demonstrate pro-resolutive and pro-efferocytosis properties.

### SuperMApo pro-resolutive factors modify APC homeostasis

During resolution of inflammation, activated APC return to homeostasis through a process called catabasis (1). When cultured with SuperMApo, TLR-activated mouse pDC, conventional DC (cDC) and macrophages demonstrated a faster return to homeostasis, as attested by an accelerated loss of co-stimulatory and major histocompatibility class (MHC)-II molecule expression (Figure 1G and Supplementary Figure 1). Whereas SuperMApo did not increase CD86, CD40 and IA/IE maturation marker expression on fresh APC (Figure 1G), it conferred pro-Treg properties to pDC and macrophages, but not cDC (Figure 1H). This was observed at the expense of Th1 polarization from naïve CD4^+^ T cells (Figure 1H), and associated with the strong decrease in inflammatory cytokine production by pDC and macrophages, notably TNF, an increase of TGF-β production (Supplementary Figure 2A). Lower IL-12 and higher TGF-β production was observed for cDC after culture with SuperMApo (Supplementary Figure 2A). This was confirmed *ex vivo* with pDC and macrophages, but not with cDC, isolated 48 h after SuperMApo injection from naïve mice, which demonstrated pro-Treg properties (Supplementary Figure 2B). When APC were cultured with SuperMApo before TLR stimulation, a reduced expression of co-stimulatory and MHC-II molecules was observed, and pDC and macrophages demonstrated pro-Treg properties (Supplementary Figure 2C). Indeed, when such APC were then sorted and cultured in the presence of naïve T cells for 5 days, pDC and macrophages strongly favored Treg cell polarization of naïve T cells in an ovalbumin-specific reactivity context (ovalbumin-charged APC plus sorted-CD4^+^CD25^−^ T cells from OTII/RAG mice). Altogether, our data demonstrated that SuperMApo factors accelerate APC catabasis and provide pDC and macrophages with pro-tolerogenic properties *in vitro* and *in vivo*. When T cell functions were assessed in the presence of SuperMApo, a strong reduction of proliferation was observed after CD3 or antigenic stimulation, associated with Treg induction (Figures 1I–K and Supplementary Figures 2D–E). These data show that SuperMApo favors Treg induction directly and indirectly through the reprograming of pDC and macrophages *in vivo* and *in vitro*.

### SuperMApo injection induces a rapid termination of ongoing collagen-induced arthritis

**Figure 2:**
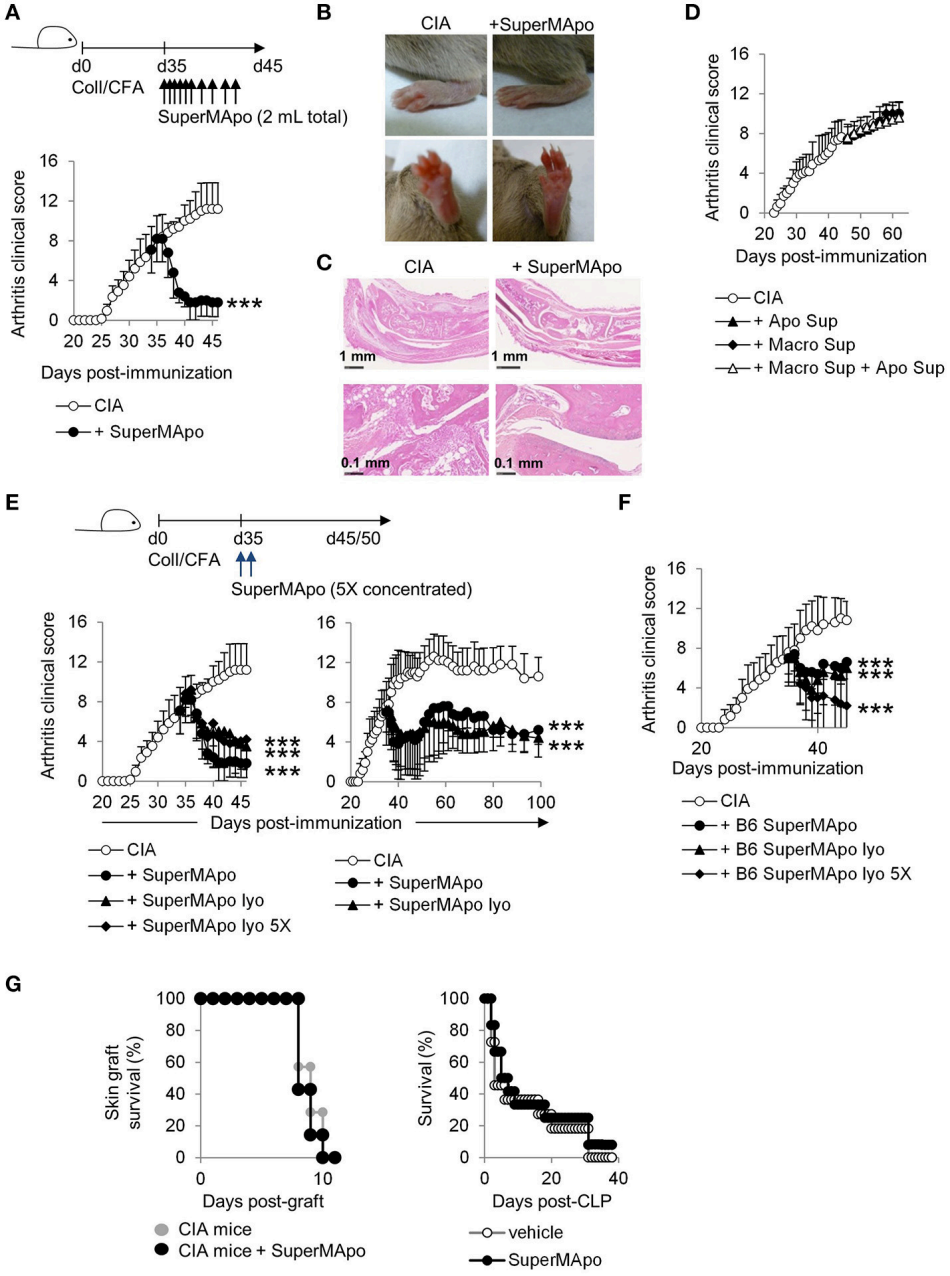
Injection of SuperMApo in ongoing CIA induced a rapid termination of the disease. **(A)** The clinical arthritic score (mean ± s.e.m., *n* = 5 per group) after SuperMApo injections was strongly reduced in arthritic mice compared to CIA mice receiving control vehicle injections (CIA). ***P < 0.001, one-way ANOVA with Bonferroni's multiple comparison post-tests. **(B)** Representative pictures of paw swelling reduction after SuperMApo injection. **(C)** HES staining of rear ankle joint sections showing vehicle-(CIA) and SuperMApo-treated arthritic mice conditions. **(D)** The clinical arthritis score (mean ± s.e.m., *n* = 5 per group) of mice receiving the supernatant from apoptotic cell culture, or from macrophage culture or both the previous together did not demonstrated reduction. **(E,F)** Arthritic mice treated with lyophilized SuperMApo concentrated (SuperMApo lyo 5X) or not (SuperMApo lyo), demonstrated arthritis clinical score reduction, lasting for more than 60 days, even when issued from a C57Bl/6 (B6) origin. ***P < 0.001, one-way ANOVA with Bonferroni's multiple comparison post-tests, vs. CIA (mean ± s.e.m., *n* = 5 per group). **(G)** SuperMApo-treated arthritic mice were able to reject an allogeneic skin graft as control mice did (7 mice per group, data pooled from 2 independent experiments), and naive mice receiving SuperMApo or vehicle demonstrated similar survival rate after cecal ligation and puncture-induced sepsis (11 to 12 mice per group, data pooled from 2 independent experiments). Figure data are issued from representative experiments, repeated at least three times with similar results unless otherwise noted.

SuperMApo (10 injections) was injected in arthritic (CIA) mice, when they exhibited an average arthritis clinical score of 7 out of 16, which corresponds to moderate/high arthritic symptoms. Soon as after the first injections, the clinical score significantly decreased (Figure 2A), from swelling red paws to healing paws (Figure 2B). Histological analysis confirmed the absence of infiltrating inflammatory cells within the joints and demonstrated neo-cartilage formation (Figure 2C). This therapeutic effect was observed only with the injection of the factors issued from efferocytosis (i.e., SuperMApo), and not with the factors issued from the culture of macrophages alone, or with the factors obtained from cultured apoptotic cells alone, or with both supernatants injected together (Figure 2D). These data clearly demonstrate that only factors issued from macrophages eliminating apoptotic cells are able to terminate ongoing chronic inflammation. Arthritis was definitively controlled by SuperMApo injection, up to 60 days after 2 injections of 5 times concentrated (using lyophilisation) SuperMApo (Figure 2E). Concentrated SuperMApo was then used. SuperMApo produced with cells from C57Bl/6 mouse strain also favored arthritis resolution in mice from the DBA/1 background, showing that SuperMApo resolves inflammation across MHC barriers (Figure 2F). Of importance, SuperMApo did not induced an over-immunosuppression, since mice receiving SuperMApo treatment were still competent to both reject an allogenic skin graft and control caecum ligation/puncture (CLP)–induced sepsis (Figure 2G). So far, SuperMApo treatment definitively terminates ongoing chronic inflammation without inducing immunosuppression.

### SuperMApo-induced resolution involves auto-antigen-specific regulatory T cell induction

**Figure 3:**
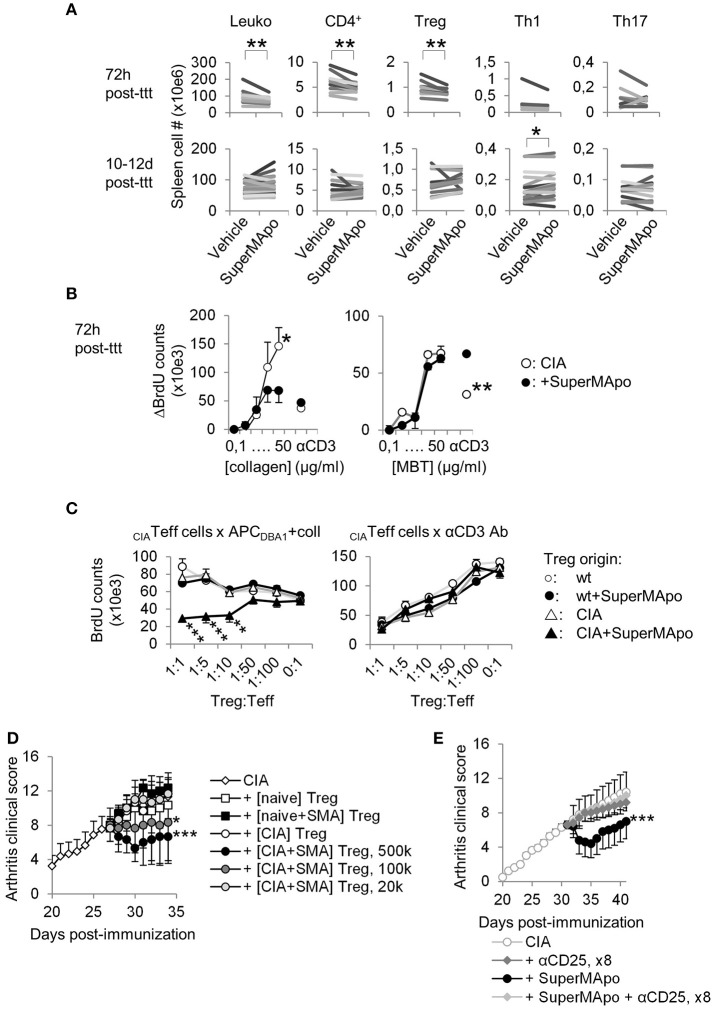
Resolution of arthritis implicates the generation of antigen-specific regulatory T cells. **(A)** Numbers of splenocytes (Leuko), CD4^+^ T cells and Treg, IFN-γ^+^ Th1 and IL-17A^+^ Th17 subsets were decreased early after SuperMApo treatment (72 h) and were observed normalized 10–12 days post-treatment (10–12 days post-ttt), compared to arthritic mice receiving vehicle. Each bar represents means of each group per one experiment with 5 mice per group. **P < 0.01, paired *t*-test. **(B)** BrdU counts showing the proliferation of cells from arthritic mice 72 h after receiving SuperMApo or vehicle, in response to increasing doses of collagen or MBT, or to CD3-specific antibody (used as control). Data are shown as mean ± s.e.m. of mouse spleen cells performed in triplicates, 5 mice per group, *P < 0.05, **P < 0.01, 2-way ANOVA with Bonferroni post-tests. **(C)** BrdU counts showing collagen-specific suppression of cell proliferation by Treg issued from arthritic mice treated with SuperMApo 72 h earlier compared to Treg from other origins. The suppressive activity of all Treg is also shown as control in CD3-specific antibody-stimulated cultures. Data are shown as mean ± s.e.m. of mouse spleen cells performed in triplicates, 5 mice per group, ***P < 0.001, 2-way ANOVA with Bonferroni post-tests. **(D)** Arthritis clinical score showing that only Treg issued from arthritic mice treated with SuperMApo ([CIA + SMA] Treg) demonstrated the capacity to reduced arthritis after adoptive transfer compared to Treg from other origins (from CIA mice [CIA], naïve mice receiving SuperMApo [naïve+SMA] or not [naïve]), in a cell number-dependent manner. Five mice per group, mean ± s.e.m., *P < 0.05, ***P < 0.001 vs. CIA group, one-way ANOVA with Bonferroni's multiple comparison post-tests. **(E)** Arthritis clinical score showing that CD25-specific depleting antibody injection inhibited SuperMApo-induced arthritis resolution. Five mice per group, mean ± sem, ***P < 0.001 vs. other groups, one-way ANOVA with Bonferroni's multiple comparison post-tests.

Three days after arthritis resolution by SuperMApo, a strong reduction of the spleen cellularity was observed (Figure 3A), which reached 38% of cell number reduction in some experiments. Total numbers of T cells, CD4^+^ T cells and Treg were decreased but not CD8^+^ T cell number (Figure 3A and data not shown). T cell percentages were not affected (Supplementary Figure 3A) and no increase of cell apoptosis after SuperMApo treatment was detected (data not shown). Later (10 to 12 d after treatment), spleen cell numbers in SuperMApo-treated mice reached those observed in age-matched untreated CIA mice, and the percentage of Treg was significantly increased (Figure 3A and Supplementary Figure 3A). In addition, 72 h and 10–12 days post-treatment a strong reduction of T cell proliferation was observed *ex vivo* in response to collagen restimulation (Figure 3B and Supplementary Figure 3B). This reduction was restricted to collagen stimulation and not to *Mycobacterium tuberculosis* (MBT) stimulation (which was mixed with collagen in the complete Freund's adjuvant for arthritis induction) (Figure 3B), demonstrating that SuperMApo induced an auto-antigen-specific immunomodulation. Because pathogenic CD4^+^ T cell numbers (Th1 or Th17) were not affected, antigen-specific Treg were evaluated using antigen-specific suppressive assays. Only Treg isolated from arthritic mice receiving SuperMApo were able to suppress collagen-specific proliferation but not Treg issued from untreated arthritic mice, nor Treg issued from naïve mice receiving or not SuperMApo (Figure 3C). When MBT was used as antigen, no suppression was observed (Supplementary Figure 3C). When CD3-specific antibody-stimulation was used, all Treg demonstrated a suppressive activity (Figure 3C). The antigen specificity of Treg induced by SuperMApo treatment in CIA mice was further confirmed using a transfer experiment in which again only the Treg isolated from arthritic mice receiving SuperMApo were able to suppress ongoing arthritis, in a dose dependent manner (Figure 3D). Furthermore, depletion of CD25^+^ cells starting the day before SuperMApo treatment inhibited SuperMApo-induced arthritis resolution (Figure 3E). Altogether, these data demonstrate that SuperMApo injection in ongoing chronic inflammation induces a brief cell contraction followed by the emergence of auto-antigen-specific Treg sustaining inflammation resolution.

### Arthritis resolution by SuperMApo initiates antigen-presenting cell reprogramming

**Figure 4:**
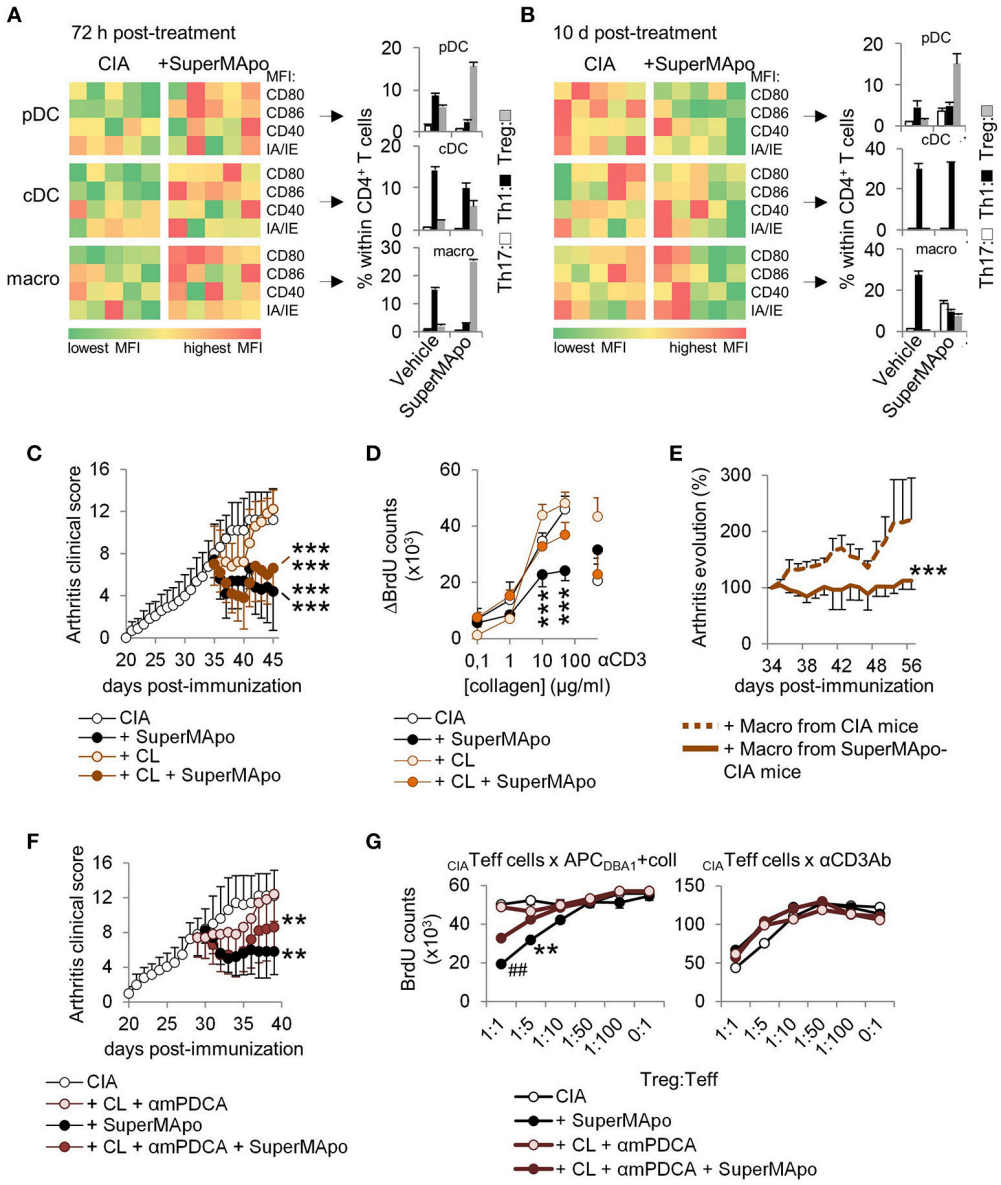
Antigen-presenting cells demonstrate reprogramming *in vivo* after SuperMApo treatment of CIA mice. **(A,B)** Costimulatory (CD80/CD86/CD40) and MHC-II molecule (IA/IE) mean florescence intensity (MFI) expressions, evaluated in spleen plasmacytoid DC (pDC), conventional DC (cDC) and macrophages (macro) 72 h and 10 days after SuperMApo or vehicle treatment of arthritic mice. Data from representative experiments showing cell marker expression from individual mouse (5 mice per group). Th17, Th1 and Treg CD4^+^ T cell polarizations by the same APC are also shown (bars represent mean ± s.e.m. of triplicates of APC isolated from each mouse and cultured with naïve CD4^+^ T cells). **(C)** Arthritis clinical score of mice receiving SuperMApo treatment or vehicle and clodronate-loaded (CL) or control liposomes (mean ± s.e.m., 5 mice per group). ***P < 0.001 vs. respective control groups (CIA & CIA + CL), one-way ANOVA with Bonferroni's multiple comparison post-tests. **(D)** BrdU counts showing the proliferation of cells from arthritic mice 72 h after receiving SuperMApo or vehicle and phagocyte depletion, in response to increasing doses of collagen or to CD3-specific antibody (as control). Data are shown as mean ± s.e.m. of cell triplicates, 5 mice per group, ***P < 0.001, 2-way ANOVA with Bonferroni post-tests. **(E)** Evolution of arthritis clinical score (in percentage; 100% = arthritis clinical score the day of injection) in mice with ongoing arthritis (mean arthritis clinical score = 7.1 ± 0.6 the day of cell injection) after receiving macrophages from vehicle- or SuperMApo-treated CIA mice. Percentages of clinical score are shown as mean ± s.e.m., 8 mice per group from 2 independent experiments. ***P < 0.001, Wilcoxon signed rank test. **(F)** Arthritis clinical score of mice receiving SuperMApo treatment or vehicle with or without clodronate-loaded liposomes and anti-mPDCA antibody (CL + αmPDCA) (mean ± s.e.m., 5 mice per group). **P < 0.005 for CIA vs. CIA + SuperMApo & CIA vs. CIA + CL + αmPDCA + SuperMApo, one-way ANOVA with Bonferroni's multiple comparison post-tests. **(G)** BrdU counts showing collagen-specific suppression of cell proliferation by Treg issued from arthritic mice from **F**. The suppressive activity of all Treg is also shown as control in CD3-specific antibody-stimulated cultures. Data are shown as mean ± s.e.m. of mouse spleen cells performed in triplicates, 5 mice per group, ^*##*^*P* < 0.005 vs. CL+mPDCA and CIA, **P < 0.005 vs. CIA, 2-way ANOVA with Bonferroni post-tests. Figure data are issued from representative experiments, repeated at least three times with similar results unless otherwise noted.

Three days after SuperMApo treatment, pDC, cDC and macrophages from treated mice were more mature compared to those from untreated arthritic mice, and demonstrated a higher capacity to favor Treg commitment from naïve CD4^+^ T cells rather than Th1/Th17 polarization (Figure 4A). Ten days after treatment, while APC were less mature than in control mice, pDC and macrophages, but not cDC, conserved a pro-Treg activity (Figure 4B), demonstrating macrophages and pDC pro-Treg reprogramming. Interestingly, despite reprograming, APC conserved the capacity to respond to TLR stimulation (Supplementary Figures 4A,B). To further address the role of pDC and macrophages in SuperMApo-induced arthritis resolution, pDC were depleted before and after SuperMApo treatment in CIA mice. Resolution of arthritis was conserved in absence of pDC together with a preserved immunomodulation to collagen auto-antigen (Supplementary Figures 4C,D), demonstrating the dispensable role of pDC. Furthermore, the transfer of pDC from SuperMApo-treated CIA mice into untreated CIA mice did not modify the course of arthritis (Supplementary Figure 4E). In contrast, when clodronate liposome-sensitive phagocytes were depleted, the clinical score was still reduced by SuperMApo treatment, but the immunomodulation to collagen was loosed (Figures 4C,D). These data suggest that macrophages are dispensable for SuperMApo-induced inflammation resolution, but are mandatory to generate tolerance to collagen antigen. In addition, the transfer of macrophages from SuperMApo-treated CIA mice into untreated CIA mice allowed a significant reduction of arthritis (Figure 4E). When both macrophages and pDC were absent, arthritis clinical score was shortly modulated by SuperMApo treatment (Figure 4F), and auto-antigen specific Treg were strongly reduced (Figure 4G). Collectively, these data demonstrate that SuperMApo treatment induces a pro-tolerogenic reprogramming of pDC and macrophages promoting auto-antigen-specific Treg and long-term resolution.

### TGF-β associated with other co-factors within SuperMApo demonstrates pro-resolutive properties

**Figure 5:**
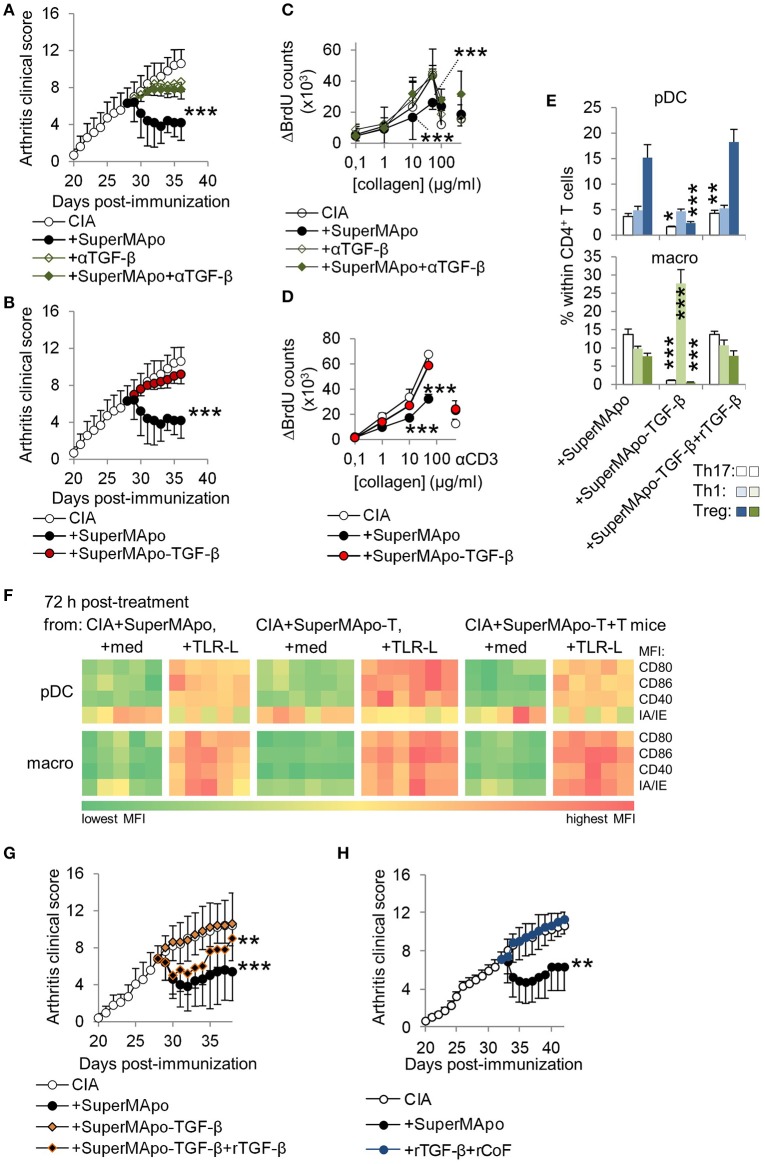
TGF-β associated with soluble factors within SuperMApo demonstrates pro-resolutive properties. **(A,B)** Arthitis clinical scores showing the role of TGF-β within SuperMApo on arthritis evolution through TGF-β neutralization (+αTGF-β) or depletion (-TGF-β) from SuperMApo. ***P < 0.001 vs. CIA (5 mice per group, mean ± s.e.m.), one-way ANOVA with Bonferroni's multiple comparison post-tests. **(C,D)** BrdU counts showing the proliferation of cells from arthritic mice from panels **A,B**. Data are shown as mean ± s.e.m. of mouse spleen cells performed in triplicates, 5 mice per group, ***P < 0.001, 2-way ANOVA with Bonferroni post-tests. **(E)** IL-17A^+^ Th17, IFN-γ^+^ Th1 and Treg CD4^+^ T cell polarization by pDC or macrophages isolated from mice from **B** and/or **G** (bars represent mean ± s.e.m. of triplicate of APC isolated from each mouse and cultured with naïve CD4^+^ T cells). *P < 0.05, **P < 0.01, ***P < 0.001, 1way ANOVA with Bonferroni's multiple comparison test. **(F)** Costimulatory (CD80/CD86/CD40) and MHC-II molecule (IA/IE) mean florescence intensity (MFI) expressions, evaluated in pDC and macrophages (macro) from spleens from mice from **B** and/or **G** (-T = SuperMApo depleted for TGF-β; -T+T = -T plus recombinant TGF-β). Data from a representative experiment showing cell marker expression from individual mouse (5 mice per group). **(G)** Arthritis clinical score showing no arthritis reduction with SuperMApo depleted from TGF-β (+SuperMApo-TGF-β) compared to SuperMApo treatment, and a partial effect of recombinant TGF-β injection on arthritis resolution when injected with SuperMApo depleted from TGF-β (+SuperMApo-TGF-β+rTGF-β). **P < 0.01, ***P < 0.001 vs. control groups (5 mice per group, mean ± s.e.m.), one-way ANOVA with Bonferroni's multiple comparison post-tests. **(H)** Arthritis clinical score showing that injection of recombinant TGF-β in addition to recombinant cofactors MMP2, ApoE, C1q, Ttr and Thbs1 (+rTGF-β+rCoF), does not favor resolution when compared to SuperMApo injection. **P < 0.01, vs. control and +rTGF-β+rCoF groups (5 mice per group, mean ± s.e.m.), one-way ANOVA with Sidak's multiple comparisons test; representative from 2 independent experiments.

Because SuperMApo favored regulatory T cell emergence, as well as pro-regulatory properties to pDC and macrophages, we thought about using first recombinant TGF-β1 (rTGF-β1) to mimic SuperMApo-induced arthritis resolution. Indeed, TGF-β was quantified in higher quantities in SuperMApo compared to control supernatants from macrophage or apoptotic cell cultures (Supplementary Figure 5A). As a major player in efferocytosis-induced immunomodulation and in tolerance induction and maintenance (17–19), *in vitro* TGF-β depletion from SuperMApo or its neutralization using a blocking antibody inhibited SuperMApo-induced APC catabasis and SuperMApo-induced reduced T cell proliferation and Treg induction (Supplementary Figure 5B–D). *In vivo*, blockade or depletion of TGF-β in SuperMApo inhibited SuperMApo-induced arthritis resolution, Treg induction and so auto-antigen-specific tolerance (Figures 5A–D and Supplementary Figure 5E). In addition, absence of TGF-β inhibited pDC and macrophage reprogramming *in vivo*, which were no more able to favor Treg commitment *ex vivo* (Figures 5E,F). Interestingly, rTGF-β injection was not able to enhance neutrophil influx during peritonitis, increase apoptotic cell phagocytosis by macrophages (data not shown) and circumvent ongoing arthritis when injected in the same quantity than detected in SuperMApo (Supplementary Figure 5F). However, when rTGF-β was combined to TGF-β-depleted SuperMApo, a short-term reduction of arthritis clinical score was observed, as well as pDC and macrophage reprogramming (Figures 5E–G). The data suggest that TGF-β plays a major role in SuperMApo-induced resolution and that it needs other factors. Other resolutive chemokines (RANTES/CCL5, MIP2/CXCL2, MDC/CCL22), and cytokines IL-1RA and IL-10 were quantified in SuperMApo (Supplementary Figure 5A) and thus added to recombinant TGF-β (all in similar quantity than measured in SuperMApo) to treat arthritis. When these 6 factors were injected (even when used 3 times concentrated) no resolution of arthritis was observed (Supplementary Figure 5G). Therefore, we then performed surface plasmon resonance experiments using biochips coated with anti-TGF-β1 antibody to identify TGF-beta cofactors. SuperMApo was then injected and mass spectrophotometry analysis of the factors retained at the bioship surface revealed apolipoprotein E (ApoE), complement component C1q, macrophage metalloelastase MMP12, thrombospondin-1 (Thbs1) and transthyretin (Ttr) as TGF-β-associated factors. Latency-associated peptide (LAP) was not detected and latent-transforming growth factor beta-binding protein 3 (Ltbp3) only inconsistently. When biochips were coated with anti-LAP antibody, ApoE, MMP12, Thbs1 and Ttr were also identified in addition to Annexin-A2 and lipoprotein lipase. We therefore injected into arthritic mice recombinant TGF-β with MMP2, ApoE, C1q, Ttr and Thbs1 but with no success, we did not observed arthritis resolution compared to SuperMApo injection (Figure 5H). These data demonstrate that SuperMApo contains multiple factors acting together to terminate inflammation. This includes TGF-β which requires additional co-factors to play a major role in SuperMApo-induced inflammation resolution.

### Factors from monocyte-derived macrophages eliminating apoptotic cells demonstrate pro-resolutive properties

**Figure 6:**
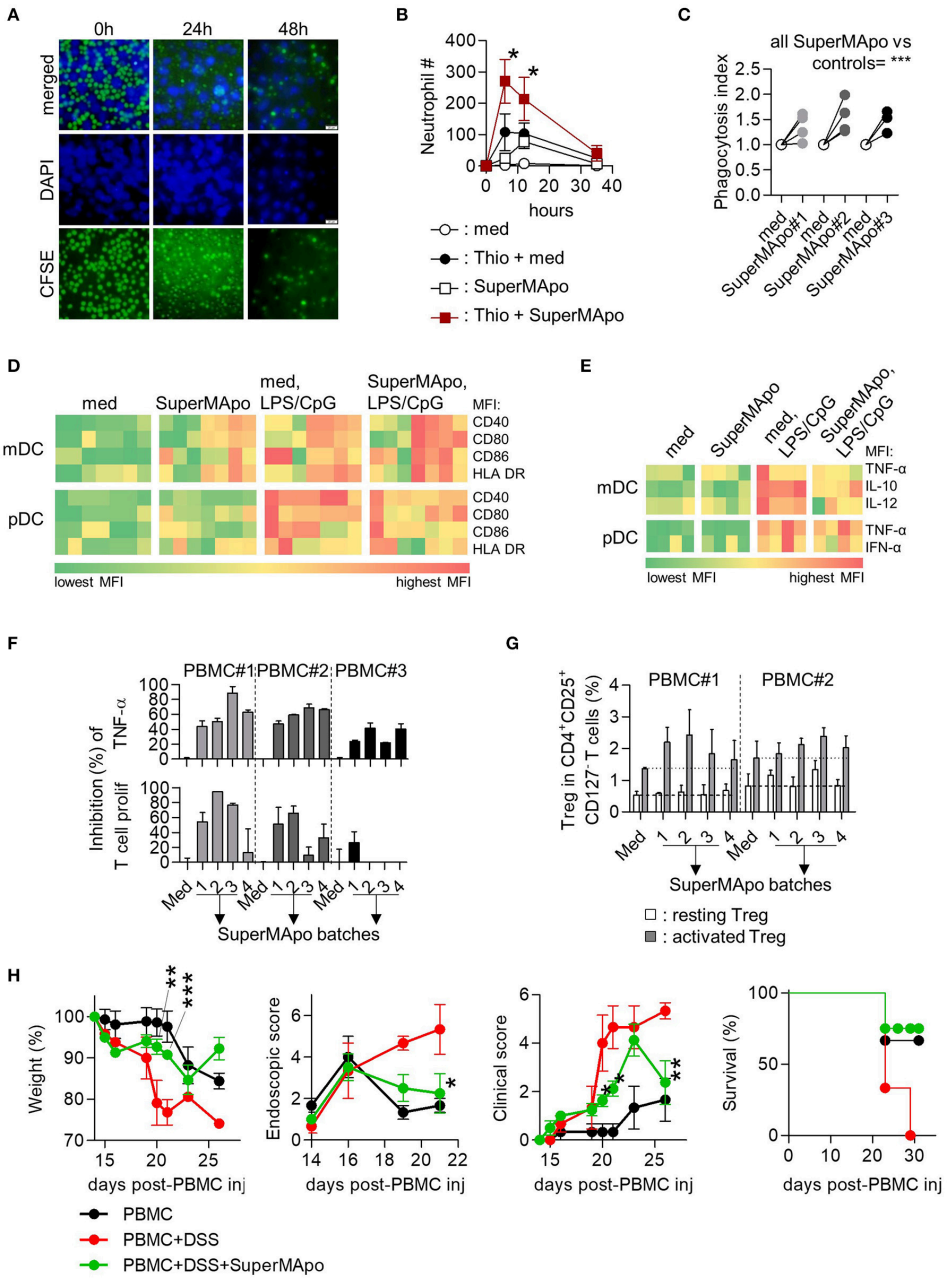
Efferocytosis factors issued from monocyte-derived macrophages demonstrate pro-resolutive properties *in vitro* and *in vivo*. **(A)** Monocyte-derived macrophages were cultured with CFSE-labeled apoptotic PBMC (green) and observed after DAPI staining by inverted fluorescence microscopy at 0, 24, and 48 h of culture. **(B)** Injection (i.p.) of the supernatant from the previous 48 h culture (SuperMApo) increased neutrophil number (mean ± s.e.m., *n* = 2-9 mice per group), 12 h after thioglycollate mobilization (Thio + SuperMApo) and control PBMC/neutrophils-reconstituted NSG mice (SuperMApo). *P < 0.05, Kruskal-Wallis test with Dunn's MCT. **(C)** Monocyte-derived macrophages demonstrated increased capacity to phagocytose apoptotic cells in the presence of SuperMApo compared to medium (med). Data from 4 individual healthy volunteers. ***P < 0.001, paired t test. **(D)** Costimulatory (CD40/CD80/CD86) and HLA-DR molecule mean florescence intensity (MFI) expressions evaluated in sorted myeloid DC (mDC) and pDC in culture with or without TLR ligands (TLR-L) or SuperMApo, or medium (med). **(E)** Intracellular cytokine percentages evaluated in sorted myeloid DC (mDC) and pDC as in **D**. One square represents the mean of duplicate obtain for cells from one healthy volunteer (n = 4 to 7), several SuperMApo batches have been used. **(F)** SuperMApo inhibition of TNF-α production was evaluated intracellularly by flow cytometry in PBMC monocytes stimulated by PHA as well as T cell proliferation within PHA-stimulated PBMC also evaluated by flow cytometry on CFSE^+^CD3^+^ T cells. PBMC were issued from different healthy volunteers (*n* = 3) and SuperMApo from different batches. Bars represent means ± s.e.m. of duplicate. (**G**) Foxp3^+^CD25^high^CD45RA^−^ activated and Foxp3^int^CD25^int^CD45RA^+^ resting Treg in CD4^+^CD127^−^ T cells were evaluated in PBMC cultured with CD3-specific antibody with or without SuperMApo. Bars represent means ± s.e.m. of triplicate. Dash lines represent Treg levels obtained in medium condition. **(H)** SuperMApo was injected in PBMC-reconstituted NOG mice receiving DSS (PBMC+DSS+SuperMApo) and then, the weight was evaluated in these mice and controls (PBMC; PBMC+DSS), as well as colonic lesion by video-colonoscopy, the clinical score was determined as well as survival. The data are shown as mean ± s.e.m. for each group, with 3–4 mice per group. *P < 0.05, **P < 0.01, ***P < 0.001, vs. DSS, 2-way RM ANOVA with Bonferroni post-tests.

In the human context, we used M2 macrophages derived from monocytes because they demonstrated efficient phagocytic capacities (Supplementary Figures 6A,B), to generate 48 h culture SuperMApo with apoptotic cells from the same donor (Figure 6A). This was injected into PBMC-reconstituted immunodeficient mice with thioglycollate-induced peritonitis and resolution was enhanced compared to vehicle injection (Figure 6B). Increased neutrophil migration by SuperMApo was confirmed *in vitro* using trans well assays (Supplementary Figure 6C). Because neutrophil number was reduced to normal levels at peritonitis resolution, the phagocytic properties of macrophages were addressed *in vitro* and we observed enhanced phagocytic capacities of monocyte-derived macrophages in the presence of human SuperMApo (Figure 6C). So far, the factors produced by human macrophages eliminating apoptotic cells enhance peritonitis resolution increasing neutrophil influx and macrophage phagocytic capacities. Human SuperMApo induced a slight maturation of myeloid DC and pDC in culture, strongly inferior to what observed with TLR ligands, and did not inhibit LPS-induced mDC maturation but limited CpG-induced pDC maturation (Figure 6D). However, mDC and pDC demonstrated decreased intracellular levels of inflammatory cytokines after TLR stimulation in the presence of SuperMApo (Figure 6E). PBMC also demonstrated a lower TNF-α content when cultured with PHA and SuperMApo compared to medium, and this was accompanied by both a lower T cell proliferation and a Foxp3^+^ Treg increase within PBMC (Figures 6F,G). Human SuperMApo was then challenged in inflammatory models of xenogeneic colitis and xenogeneic graft-*vs*.-host disease (GvHD). Treatment with 3 time-concentrated SuperMApo strongly limited colitis severity, as attested by weight loss reduction and a strong decrease of colonic lesions as observed by video-endoscopy, associated with colitis clinical score reduction and survival improvement (Figure 6H). In xenogeneic GvHD, 5-time concentrated SuperMApo treatment also strongly limited GvHD clinical score and improved survival (Supplementary Figure 6D). Altogether, our data demonstrated that human macrophages eliminating apoptotic cells produce resolutive factors able to control acute and chronic inflammation.

## Discussion

Here, we provide data showing that re-introduction of the factors issued from efferocytosis are able to treat acute and chronic inflammation. We also show that the resolution of inflammation by SuperMApo (i.e., efferocytosis factors) implicates the reprogramming of macrophages and pDC. Those macrophages sustain a long-term control of inflammation through the induction of auto-antigen-specific Treg. Additionally, we demonstrate that human efferocytosis factors are also able to control ongoing inflammation in xenogeneic experimental models. This highlights SuperMApo as a new therapeutic option in the treatment of acute and chronic inflammatory diseases.

The following conclusions can be drawn from the current study. First, the factors issued from apoptotic cell elimination by macrophages not only control the auto-antigen-specific response, but also induce long-term tolerance to the pathogenic auto-antigen. Other therapeutic approaches, inspired from the resolution process, have been proposed and notably injection of apoptotic cells (2, 6). In experimental models of inflammation, however, apoptotic cell injection never induced long term tolerance, (2, 6, 20) and in particular, in the treatment of CIA (21). Tolerance is reached in models of inflammation using apoptotic cell mimicry with antigen-coupled apoptotic cell injection (22, 23), or using antibody injection inducing cell apoptosis *in vivo*, as well in the presence of the pathogenic antigen (24, 25). Here, our data demonstrate that SuperMApo treatment precipitates termination of ongoing arthritis together with an auto-antigen-specific Treg induction. Because Treg induction has been also observed in other apoptosis-based resolutive approaches, (23–27) our data suggest that Treg might be only necessary to maintain tolerance but not sufficient to terminate inflammation.

Second, the tolerance induced by SuperMApo treatment is restricted to the pathogenic autoantigen, collagen, and does not spread to other antigens. CD4^+^ T cells isolated from SuperMApo-treated CIA mice demonstrated a normal response to polyclonal stimulation, suggesting the absence of overall immunosuppression. This is further supported by the fact that SuperMApo-treated CIA mice strongly rejected allogeneic skin graft. More interestingly, during the resolution of inflammation triggered by SuperMApo, tolerance is not extended to bacterial antigen (MBT), nor to the microbiota since SuperMApo-treated mice demonstrated similar survival than naïve mice after sepsis induced by CLP. Thus, infectious and allogeneic antigens are discriminate from self-antigens and therefore do not induced similar inflammation and subsequent resolution.

Finally, our data demonstrate that taken as a whole, factors issued from efferocytosis act in synergy to resolve inflammation. Latency-associated protein (28), GARP (29) or α_v_β_8_ integrin (30) have been demonstrated to be implicated in TGF-β activity. Our data provide new actors which might extend the stability of TGF-β active complex or its signaling rather than TGF-β activity. Indeed, within SuperMApo, TGF-β cooperates with ApoE, C1q, MMP12, Thbs1, Ttr, Annexin A2, and lipoprotein lipase, maybe, to enhance its stability, distribution and bioavailability. Further work is necessary to confirm such findings, and might provide complexed TGF-β formulation with a high clinical efficacy.

In summary, our work demonstrates a new way of research and development to treat inflammatory diseases by rebooting resolution to terminate ongoing inflammation. The cell-free complex biological drug SuperMApo has also demonstrated a significant therapeutic effect to terminate experimental Crohn disease (OMR, TG, FB, AM, PS, MC and SP, in preparation) and to stabilize autoimmune encephalomyelitis (TG, OMR, FB, AM, PS, MC and SP, in preparation), and is now under clinical development. Moreover, while SuperMApo opens a new insight to manage inflammatory diseases, our work also highlights the process of resolution as a main axis of research to identify innovative therapeutic approaches.

## Online methods

### Mice

Male or female DBA/1 (Janvier; Le Genest-Saint-Isle, France), C57Bl/6 (Charles River), OTII/RAG^−/−^ C57Bl/6, NOG and NSG (Charles River) mice age 10–12 weeks were housed in filter-top cages with freely available food and sterile water (Plexx), at the UMR1098 Animal facility (agreement #C25-056-7). NOG mice were originally provided by the central institute for experimental animals. All experimental studies complied with European legislation and were approved under project number #02831 by local (Animal ethic committee of Besançon [Comité d'Ethique Bisontin en Experimentation Animale], #58) and national (French Ministry of Higher Education and Research [Ministère de l'Education Nationale, de l'Enseignement Supérieur et de la Recherche]) authorities for the care and use of animals.

### Efferocytosis

To perform efferocytosis, mouse thymic cells were used as apoptotic cells. After isolation through mushing, thymic cells were submitted to a 35 X-Gray irradiation (Raycell blood irradiator; Best Theratronic) and cultured for 6 h to allow apoptosis to occur (21, 26, 31) in Dulbecco Modified Eagle Medium supplemented with 10% heat-inactivated fetal calf serum (FCS; Life Technologies), 1% penicillin/streptomycin, 10 mM HEPES buffer (Sigma Aldrich), 10 mM nonessential amino acids (Invitrogen), and 0.05 mM 2-mercaptoethanol (Sigma Aldrich). Apoptosis was confirmed by flow cytometry using positive staining for Annexin-V and 7-AAD exclusion (BD Bioscience). In parallel, macrophages were isolated from the peritoneum cavity 48 h after thioglycollate mobilization (3%, 1 ml/mouse i.p.), washed and cultured in completed RPMI (10% heat-inactivated FCS, 1% penicillin/streptomycin, 10 mM HEPES buffer [Sigma Aldrich], 10 mM nonessential amino acids [Lonza], 4 mM L-glutamine [Biowhittaker], 1 mM sodium pyruvate [Sigma Aldrich], and 0.05 mM 2-mercaptoethanol) for 6 h. Macrophage enrichment was attested by flow cytometry using anti-CD11b (clone M1/70; BD Biosciences) and anti-F4/80 antibody staining (clone B18; eBioscience). Macrophages and apoptotic cells were then washed and cultured together for 48 h in phenol-free X-VIVO15 (Lonza) to a 1 macrophage for 5 apoptotic cell ratio. Supernatant (=SuperMApo) containing pro-resolutive mediators was then collected, centrifuged to eliminate debris, filtered through a 0.22 μm filter and frozen or lyophilized for conservation. Apoptotic cell efferocytosis was observed using regular or fluorescent inverted microscopy using CFSE-labeled apoptotic cells and CD11b-PE-labeled macrophages over time. Apoptotic cells or macrophages were cultured alone for the same period and the supernatants were collected as controls. SuperMApo was depleted from TGF-β using microbeads (Polysciences) coated with anti-mouse IgG (Fc specific) and anti-TGF-β Ab (clone 2G7) following manufacturer instructions. Human SuperMApo was generated using monocyte-derived macrophages issued from buffy coats from healthy volunteers cultured with apoptotic irradiated peripheral blood mononuclear cells from the same volunteer for 48 h. Supernatant was then collected, centrifuged to eliminate debris, filtered through a 0.22 μm filter and frozen or lyophilized for conservation.

### Animal models and treatments

Collagen-induced arthritis was induced and scored as previously described (21). Arthritis developed at day 20–25 after collagen immunization in all mice. The day of treatment, mouse SuperMApo was injected or reconstituted in distilled water and injected i.p. or i.v. in arthritic mice in 10 injections (10 times of fresh SuperMApo 200 μL every day for five injections and then every 2 days) or in two injections when lyophilized and concentrated five times (2 times 200 μL once and 48 h later). Anti-TGF-β antibody (clone 1D11 [R&D Systems] or clone 2G7 [provided by L Chatenoud; Necker Hospital, Paris, France]) was given i.p. the day of treatment (150 μg/mouse) and 48 h later (100 μg/mouse). Recombinant TGF-β (R&D Systems) was given as 1 ng/mouse (i.p.). Plasmacytoid DC depletion was performed using purified anti-mouse PDCA-1 antibody (clone JF05-1C2.4.1; Miltenyi Biotec) i.p. infusions (1 mg/mouse, 4 injections of 250 μg every 2 d, starting 3 d before treatment). Homemade (26) clodronate-loaded or PBS-loaded liposomes were injected i.v. 48 h before treatment for phagocyte depletion. For caecum ligature puncture (CLP), mice were anesthetized by isoflurane and an abdominal middle incision was made to expose caecum which was the isolated and ligated to 75% of the length of the organ with 4-0 silk suture. Then the caecum was punctured one time with 21G needle, a small amount of fecal material is extruded and the caecum is replaced in the abdominal cavity and the incision was closed with 6-0 silk suture. The animal receives an injection of 400 μl of PBS at 37°C to compensate the loss of water. Mice received 1 ml of fresh SuperMApo or control medium i.p. 24 and 72 h after the operation. Survival was recorded daily. Concerning skin allograft, donor skin was harvested from C57Bl/6J or DBA1 mouse tail and conserved on ice in PBS. Recipient mice were anesthetized and a 1 cm^2^ piece of epidermis was removed on the back side of each recipient and replace by 1 cm^2^ piece of donor skin sutured with 10-0 silk suture. About *in vivo* resolution model, C57Bl/6J mice or leukocyte-reconstituted NOG mice received an injection of 3% thioglycollate i.p. and SuperMApo (mouse or human) or control medium. NOG mice received 25.10e6 PBMC and 15.10e6 PMN 2 h before thioglycollate and SuperMApo injections. A peritoneal lavage with cold HBSS was then performed in the following hours and leucocyte content was counted and analyzed by flow cytometry. Xenogeneic colitis was induced in NSG mice by the administration of 3% of DSS (MP Biomedicals) in drinking water during 7 days, 14 days after the injection of 4.10e6 human PBMC. At days 14 and 16 some mice received 2 injections of 1 mL of 3 times concentrated SuperMApo. Colitis clinical score was monitored until day 26 by clinical and MEICS endoscopic scores as described before (32, 33). Xenogeneic GvHD was induced in NOG mice by the injection of 10.10e6 PBMC (i.v.). Ferrara' clinical score (34) (to assess GvHD severity), weight loss and survival were monitored daily.

### Cell sorting, culture, and analysis

Lymphoid organs were harvested, dissociated, and erythrocytes were removed by osmotic shock. After washing, lymphoid cells were directly used for culture or for pDC, cDC, macrophage or T cell enrichment using commercial kits (mPDCA-1 microbeads, CD11c microbeads, CD11b microbeads or CD4^+^CD25^+^ regulatory T cell isolation kit, respectively [Miltenyi Biotec]) for culture or transfer. Cell purity was assessed by flow cytometry using mPDCA (clone 927; Biolegend), CD11c (clone N418; Biolegend), siglec-H (clone eBio440e; eBioscience) and B220 (clone RA3-6B2; BD Biosciences) staining for pDC, CD11c for cDC, F4/80, and CD11b for macrophages and CD4 (clone RM4-5; BD Biosciences), CD25 (clone PC-61; BD Biosciences) and Foxp3 (clone FJK-16s; eBioscience) for naïve and regulatory T cells. Cells were incubated with SuperMApo, or medium or control supernatants for 24 or 48 h or 5 days (T cell assays). Spleen cells or isolated APC were stimulated with LPS (1 μg/mL) and CpG (12 ng/mL) for 24 h before or after culture with SuperMApo. Then, APC were stained for the expression of CD40 (clone 3/23; Biolegend), CD80 (clone 16-10A1; BD Biosciences), CD86 (clone GL-1; Biolegend), IA/IE (clone M5/114.15.2; Biolegend) and intracellular cytokines TNF-α (clone MP6-XT22; BD Biosciences), IL-6 (clone MP5-20F3; Biolegend), and IL-12 (clone C15.6; BD Biosciences). Enriched pDC, cDC and macrophage culture supernatants were also collected for ELISA quantification of TNF, IL-6, IL-12 (Biolegend) and TGF-β (Promega) using commercial kits. In some experiments, isolated APC were cultured with naïve CD4^+^CD25^−^ T cells isolated from OTII/RAG^−/−^ mice in the presence of OVA_323−339_ peptide (2 μg/mL; Invivogen) for 5 d, and T cell polarization was then analyzed by flow cytometry. After stimulation by soluble anti-CD3 antibody (0.5 μg/mL [clone 145-2C11; eBiosciences]) or CD3/CD28 Ab (2 and 0.5 μg/mL, respectively [eBiosciences]) or APC, T cells were stained for the expression of IFN-γ, IL-17 in the presence of 50 ng/mL of phorbol myristate acetate and 1 μg/mL of ionomycin and brefeldin A (1 μL/mL/10e6 cells; BD Pharmingen) for the last 4 h of culture and for Foxp3 transcription factor. Spleen cells were also incorporated with and stained for BrdU to assess cell proliferation (Perkin Elmer) in the presence of collagen, ovalbumin or mycobacterium toxin (MBT). In some experiments, naïve T cells isolated from OTII/RAG^−/−^ mice were cultured with OVA (0–10 μg/mL). Cell staining was analyzed by flow cytometry using a BD Canto II with Diva software (BD Biosciences) or a SP8600 SONY cytometer. For suppressive assays, Treg isolated by MACS from the spleen of CIA or naïve mice treated by SuperMApo or not, were added in decreasing numbers (from 1:1 to 1:100) to effector T cells (CD4^+^CD25^−^) issued from CIA mice stimulated by collagen-loaded DBA1 APC or CD3-specific Ab (0.5 μg/mL). Effector T cell proliferation was evaluated at day 5 by BrDU incorporation and count the following day.

### Human cell manipulation

Buffy coats were collected from healthy blood donors after regulatory approval of the local ethics committee of Franche-Comté (CPP Est II, Besançon, France) and the French Ministry of Higher Education and Research (*Ministère de l'Education Nationale, de l'Enseignement Supérieur et de la Recherche*, agreement number #AC-2015-2408, May 22 2015). Neutrophils (CD15^+^) were isolated from buffy coats after Ficoll-Paque (GE Healthcare Life Sciences) separation and dextran (Sigma) sedimentation of peripheral blood mononuclear cells (PBMC). PBMC were cultured in presence of PHA (10 ng/mL; Sigma) to assess T cell proliferation by carboxyfluorescein succinimidyl ester dilution on CD3^+^ T cells evaluated by flow cytometry, or in presence of LPS (10 ng/mL; Sigma) and/or CpGA (12 μg/mL; LifeTechnology) to evaluate CD14^+^ monocytes, CD14^−^CD123^+^ pDC and CD14^−^CD123^−^CD11c^+^ mDC expression of costimulatory molecules CD40, CD86, CD80, and HLA-DR (BD Biosciences and Miltenyi Biotec) by flow cytometry. Fixable viability dye staining was used to exclude dead cells. Monocyte-derived macrophages were generated from PBMC cultured 7 days in presence of M-CSF (50ng/mL, Miltenyi Biotec; M2) with or without IL-4 (40 ng/mL, Miltenyi Biotec; M2a), IL-10 (50 ng/mL, Miltenyi Biotec; M2c), IL-4/IL-10/TGF-β (50 ng/mL Peprotech; Mreg) or in presence of GM-CSF/IFN-γ (50 ng/mL, Miltenyi Biotec; M1). Human SuperMApo was generated using monocyte-derived M2 macrophages issued from buffy coats from healthy volunteers differentiated in RPMI medium supplemented with 1%PS and in presence of M-CSF (50ng/mL, Miltenyi Biotec) during a 7 d culture. After removal of non-adherent cells, macrophages were cultured with apoptotic irradiated PBMC from the same volunteer at a ratio of 1:5 in MEM medium for 48 h. Supernatant was then collected, centrifuged to eliminate debris, filtered through a 0.22 μm filter and frozen or lyophilized for conservation.

### Statistical analysis

All data were analyzed for statistical significance using GraphPad Prism version 7.04 (GraphPad Software, San Diego, CA, USA) by adapted student *t*-test or one or two-way ANOVA test including multiple comparison post-tests.

## Author contributions

SP conceived and designed the study. FB performed and analyzed the rodent experiments. TG and RV participated to rodent experimentations. OM-R realized mouse video-endoscopy. MC performed and analyzed the human experiments. AM participated to the human experiments. AD helped on all experiments. SV-D performed the histological analysis and scoring of the joints. PS and SP wrote the manuscript. SP supervised the study. All authors discussed the results and the manuscript.

### Conflict of interest statement

FB, PS, and SP co-registered the SuperMApo patent (#WO2014106666-A1). The remaining authors declare that the research was conducted in the absence of any commercial or financial relationships that could be construed as a potential conflict of interest.
