# Supplementary material for: Factors Produced by Macrophages Eliminating Apoptotic Cells Demonstrate Pro-Resolutive Properties and Terminate Ongoing Inflammation
Source: Front Immunol. 2018 Nov 13;9:2586. doi: 10.3389/fimmu.2018.02586 (PMC6277856; doi:10.3389/fimmu.2018.02586)
Supplement: Supplementary file 1 [file Data_Sheet_1.docx]

Factors produced by macrophages eliminating apoptotic cells demonstrate pro-resolutive properties and terminate ongoing inflammation

**Authors:** Francis. Bonnefoy^1^, Thierry. Gauthier^1^, Romain. Vallion^1^, Omayra. Martin-Rodriguez^1^, Anais. Missey^1^, Anna. Daoui^1^, Séverine. Valmary-Degano^2^, Philippe. Saas^1^, Mélanie. Couturier^1,3^, Sylvain. Perruche^1,3^*

**Supplementary Materials:**

**Supplementary Figures:**

**Fig. S1.** Costimulatory (CD86/CD40) and MHC-II molecule (IA/IE) mean florescence intensity (MFI) expressions were evaluated in spleen pDC, cDC and macrophages (macro) 48 h after culture with control medium (med) or TLR ligands (TLR-L; LPS for cDC and macrophages, CpG for pDC) for 24 h and with SuperMApo for additional 24 h by flow cytometry. Mean fluorescence intensity example are given in **A**, and as triplicates plus mean (black bars) in **B**. ANOVA statistical analysis results, including Turkey’s multiple comparisons post-test, are given in **C**. Data shown are from one representative experiment out of 3 giving similar results.

**Fig. S2.** Macrophages eliminating apoptotic cells produced factors with pro-resolutive properties. (**A**) The cytokines TNF, IL-6, IL-12 and TGF-β were quantified by ELISA in the milieu of plasmacytoid dendritic cells (pDC), conventional DC (cDC) and macrophages (macro) cultured with or without of SuperMApo in the presence of TLR ligands (TLR-L) or control medium (med). (**B**) The ovalbumin (OVA) TCR-specific CD4^+^CD25^–^ T cell polarization (mean + s.e.m., *n* = 3) by pDC, cDC and macrophages issued from naïve mice injected with SuperMApo or vehicle, in the presence of OVA, was assessed by FACS evaluating IFN-γ (Th1), IL-17 (Th17) and Foxp3 (Treg) intracellular content in CD4+ T cells after 4 days of culture. (**C**) Costimulatory (CD80/CD86/CD40) and MHC-II molecule (IA/IE) mean florescence intensity (MFI) expressions were evaluated in spleen pDC, cDC and macrophages (macro) 48 h after culture with SuperMApo or control medium (med) for 24 h and with TLR ligands (TLR-L) for additional 24 h by flow cytometry. The same APC were cultured with naïve T cells and T cell polarization was assessed by flow cytometry after 4 days of culture as in **B**. Data are shown as mean + s.e.m. of triplicate. APC culture supernatants were also quantified for cytokines by ELISA. Data are shown as mean + s.e.m. of triplicate. (**D**) Spleen ova-specific T cell proliferation in the presence of grading doses of ova with medium (med) or SuperMApo in different proportions was assessed by BrdU incorporation and counting. (**E**) CD4 T cell polarization from naïve CD4^+^CD25^–^ T cells cultured with ova as in **D** in medium (med) or different proportions of SuperMApo was assessed by flow cytometry.

**Fig. S3.** Resolution of arthritis by SuperMApo injection implicates the generation of autoantigen-specific regulatory T cells. (**A**) Percentages of spleen CD4^+^ T cells and Foxp3^+^ Treg, IFN-γ^+^ Th1 and IL-17A^+^ Th17 subsets from CIA mice 72 h and 10-12 d after SuperMApo or vehicle injection. Each bar represents one experiment with the mean of each group with five mice per group. *P* = 0.016, paired *t* test. (**B**) BrdU counts showing the proliferation of cells from arthritic mice 10-12 d after receiving SuperMApo or vehicle (CIA), in response to increasing doses of collagen or to CD3-specific antibody (used as control). Data are shown as mean + s.e.m. of mouse spleen cells performed in triplicates, 5 mice per group, * *P* < 0.05, *** *P* < 0.001, 2-way ANOVA with Bonferroni post-tests. (**C**) BrdU counts showing MBT-specific cell proliferation suppression by Treg issued from arthritic mice treated with SuperMApo 10-12 d earlier or Treg from vehicle-treated CIA mice. Data are shown as mean + s.e.m. of mouse spleen cells performed in triplicates, 5 mice per group.

**Fig. S4.** Antigen-presenting cells demonstrated reprogramming *in vivo* after SuperMApo treatment of CIA mice. (**A**,**B**) Costimulatory (CD80/CD86/CD40) and MHC-II molecule (IA/IE) mean florescence intensity (MFI) expressions, evaluated in isolated spleen plasmacytoid DC (pDC), conventional DC (cDC) and macrophages (macro) issued from CIA mice receiving 72 h and 10 d earlier SuperMApo or vehicle treatment, and cultured or not (+med) with TLR ligands (+TLR-L) for 24 hours. Data from representative experiments showing cell marker expression from individual mouse (5 mice per group). (**C**) Arthritis clinical score of mice receiving SuperMApo treatment or vehicle and anti-mPDCA depleting antibody (αmPDCA) to deplete pDC or isotype (mean + s.e.m., 5 mice per group). ** *P* < 0.005, *** *P* < 0.001 *vs* respective control groups, one-way ANOVA with Bonferroni’s multiple comparison post-tests. (**D**) BrdU counts showing the proliferation of cells from arthritic mice 72 h after receiving SuperMApo or vehicle and pDC depletion, in response to increasing doses of collagen or to CD3-specific antibody (as control). Data are shown as mean + s.e.m. of cell triplicates, 5 mice per group, ** *P* < 0.005 vs +αmPDCA, $=(*** *vs* CIA and αmPDCA), #=(* *vs* CIA, *** *vs* αmPDCA), 2-way ANOVA with Bonferroni post-tests. (**E**) Evolution of arthritis clinical score (in percentage; 100%=arthritis clinical score the day of injection) in mice with ongoing arthritis (mean arthritis clinical score = 7.1 + 0.6 the day of cell injection) after receiving pDC from vehicle- or SuperMApo-treated CIA mice. Percentages of evolution of the clinical score are shown as mean + s.e.m., 8 mice per group from 2 independent experiments.

**Fig. S5.** TGF-β within SuperMApo demonstrates a pro-resolutive activity when associated with other efferocytosis factors. (**A**) Quantification by ELISA of total and active TGF-β, RANTES, MIP2, IL-1RA, MDC and IL-10 in SuperMApo and control supernatants issued from apoptotic cell culture (Apo Sup) or macrophage culture (Macro Sup), given as mean + s.e.m. of 5 to 14 individual samples. (**B**) Costimulatory (CD80/CD86/CD40) mean florescence intensity (MFI) expressions, evaluated on isolated pDC, cDC and macrophages (macro) 48 h after culture with medium (med), TLR ligands (+TLR-L), TGF-β (T) and/or SuperMApo depleted or not for TGF-β (SuperMApo-T), with or without anti-TGF-β blocking antibody (SuperMApo+αT). Data from representative experiments showing cell marker expression from triplicates from one representative experiment out of 3. (**C**) Spleen T cell proliferation in the presence of grading doses of anti-CD3 specific antibody (αCD3) with medium (med) or SuperMApo depleted or not for TGF-β (SuperMApo-T), or with recombinant TGF-β (+TGF-β), was assessed by BrdU incorporation and counting. *** *P* < 0.001, *vs* med (mean + s.e.m., *n* = 3), one-way RM ANOVA with Tukey's multiple comparison test. (**D**) IL-17A^+^ Th17, IFN-γ^+^ Th1 and Treg CD4^+^ T cell polarization by pDC, cDC or macrophages from **C** (bars represent mean + s.e.m. of triplicate of APC isolated from each mouse and cultured with naïve CD4^+^ T cells). * *P* < 0.05, ** *P* < 0.01, *** *P* < 0.001, 1way ANOVA with Bonferroni's multiple comparison test. (**E**) The percentage of Treg (CD25^+^Foxp3^+^) within CD4^+^ T cells was evaluated *ex vivo* by FACS in the spleen of arthritic mice receiving or not SuperMApo (+SuperMApo), anti-TGF-β antibody (αTGF-β), SuperMApo plus αTGF-β (+SuperMApo+αTGF-β) or SuperMApo depleted from TGF-β (+SuperMApo-TGF-β). Data show individual mice plus mean (black bars), 5 mice per group, from one representative experiment. * *P* < 0.05, ** *P* < 0.01, paired t test *vs* CIA group. (**F,G**) Arthritis clinical score of mice receiving SuperMApo treatment or vehicle or recombinant TGF-β (rTGF-β; in the same quantity as quantified in SuperMApo; **F**) or the recombinant proteins RANTES, MIP2, IL-RA, MDC, IL-10 and TGF-β (recProt; also in the same quantities as quantified in SuperMApo) or 3 time more recProt (recProt 3x) (**G**) (mean + s.e.m., 5 mice per group). *** *P* < 0.001, one-way ANOVA with Bonferroni’s multiple comparison post-tests.

**Fig. S6.** Efferocytosis factors issued from monocyte-derived macrophages demonstrate pro-resolutive properties *in vitro* and *in vivo*. (**A**) The percentage of macrophages obtained from monocyte differentiation in different conditions (M1: GM-CSF+IFN-γ; M2: M-CSF; M2a: M-CSF+IL-4; M2c: M-CSF+IL-10; Mreg: M-CSF+IL-4, IL-10 and TGF-β) was evaluated by microscopy. (**B**) The same macrophages were evaluated for their phagocytic capacities of apoptotic CFSE^+^ apoptotic cells by FACS after 2 h of culture. Data are shown as mean + s.e.m. of differentiation or phagocytosis obtained from 2 to 4 individual volunteers. (**C**) The number of neutrophils migrating through a 3 µm filter was determined by flow cytometry and count after 18 h of culture in different control conditions (RPMI +/- FBS or control medium +/- FBS) or with SuperMApo +/- FBS. Data are presented as mean + s.e.m. from one representative experiment out of 3 showing similar data. 1way ANOVA with Dunn’s multiple comparison test. (**D**) The graft *vs* host disease Ferrara’ score and mice survival were determined in NOG mice receiving PBMC and treated or not (GvHD) with SuperMApo concentrated 3 times (SuperMApo batch#2 x3) or not (SuperMApo batch#1 and SuperMApo batch#2). Data are shown as mean + s.e.m. of 7 to 9 mice per group from 2 individual experiments. For Ferrara’ score: *** *P* < 0.001 (SuperMApo batch#2 x3 *vs* GvHD), Friedman test with Dunn's multiple comparison test; for survival: *** P* <0.01, Log-rank (Mantel-Cox) test.
